# Motor Band Sign in Primary Lateral Sclerosis: A Case Report Proposing the Need for an Imaging Biomarker

**DOI:** 10.7759/cureus.36121

**Published:** 2023-03-14

**Authors:** Vijaya Lakhsmi Valaparla, Milena Lobaina, Chilvana Patel, Anand Vilaschandra Patel

**Affiliations:** 1 Neurology, University of Texas Medical Branch, Galveston, USA; 2 Neurology, Baylor College of Medicine, Houston, USA

**Keywords:** motor band sign, upper motor neuron, motor neuron disease, amyotrophic lateral sclerosis, primary lateral sclerosis

## Abstract

Motor neuron disease is a degenerative condition involving both upper motor neurons (UMN) and lower motor neurons (LMN). While amyotrophic lateral sclerosis (ALS) is an overlap of upper and lower motor neuron involvement, primary lateral sclerosis (PLS) is predominantly an upper motor neuron involvement with lower motor involvement seen in the later stages of illness. Diagnostic criteria rely on clinical features and electrodiagnostic tests such as electromyography (EMG). EMG predominantly helps in determining lower motor neuron involvement. No definitive objective measures are currently available to determine upper motor neuron involvement.

We describe a patient diagnosed with PLS based on consensus diagnostic criteria. The patient had absent LMN features both clinically and on EMG. Magnetic resonance imaging (MRI) was significant for hypointense signals in the bilateral motor strip area on susceptibility weighted sequence, suggesting a surrogate marker of degeneration involving motor neurons in the brain.

Early recognition of this MRI pattern called motor band sign (MBS) can help determine the earlier diagnosis of this neurodegenerative condition, potentially translating to better treatment and outcome measures.

## Introduction

Motor neuron disease is a degenerative condition involving both upper motor neurons (UMN) and lower motor neurons (LMN). Amyotrophic lateral sclerosis (ALS) is a progressive neurodegenerative disorder that is classically defined by the clinical involvement of both upper motor neurons (UMN) and lower motor neurons (LMN). Per the Gold Coast criteria, the diagnostic criteria for ALS include progressive neurodegeneration, UMN and LMN involvement, and exclusion of mimicking diseases. Symptoms of ALS and primary lateral sclerosis (PLS) might overlap in the early stages of the disease; however, PLS tends to have early UMN involvement with a more insidious onset of LMN features. This could delay this condition’s diagnosis and treatment [1]. Although we rely on clinical features in most patients, electrodiagnostic studies such as electromyography (EMG) can help determine the involvement of LMN. Despite scientific evolution, there are no definite objective measures to determine UMN involvement and the degree of degeneration. This represents a limiting factor in the diagnosis of motor neuron disease since the clinical features of UMN degeneration might appear late in the course of the disease, thus delaying diagnosis and treatment.

Motor band sign (MBS) is a rare finding in brain imaging in motor neuron disease. MBS in ALS was initially described as T2 hypointensities that correlated with hypointensities in the susceptibility weighted image (SWI) sequence of brain magnetic resonance imaging (MRI). SWI sequence in MRI uses susceptibility differences based on the paramagnetic properties of the underlying tissues [2]. In motor neuron disease, these changes are found along the precentral cortex, which gives it the classical appearance of the motor band sign. This MBS can be considered a marker for UMN involvement. It is important to also note that T2 hypointensities are nonspecific and can be found in many conditions such as Alzheimer’s disease, Parkinson’s disease, spinocerebellar ataxia 17, multiple cerebral infarctions, and normal aging. These findings are nonspecific to any particular disease process and need not be necessarily found on SWI sequences [3].

A retrospective study conducted by Chung et al. [4] has compared the frequencies of motor band signs in ALS, primary lateral sclerosis (PLS), and healthy controls, concluding that MBS is a surrogate marker of UMN involvement in motor neuron disease. A previous study by Roeben et al. [5] demonstrated the same finding of motor band sign being found in up to 80% of patients diagnosed with ALS who had imaging with SWI sequence being included in the protocol. Severe LMN involvement with muscular atrophy can potentially mask the findings of UMN involvement in ALS. A motor band sign was found in one such reported patient [6], suggesting the utility of this sign in identifying UMN involvement in the scarcity of clinical features of such involvement.

With the advent of new imaging technologies and the understanding of features associated with different sequences, MBS can serve as a potential surrogate marker of UMN involvement in ALS and PLS. In this context, we present a patient with diagnosed PLS and motor band sign on MRI.

## Case presentation

Our patient is a 78-year-old right-handed female with a past medical history of hyperlipidemia and a former smoker. She initially presented to the neuromuscular clinic for evaluation of dysarthria and progressive muscle weakness. Her symptoms started 10 years prior to her presentation. Her weakness initially started in the lower extremities, with the right side worse than the left. Weakness gradually progressed, leading to falls and needing a rolling walker. Over the next two years, her weakness progressed to bilateral upper extremities. She also experienced muscle stiffness in the lower extremities. Additionally, she has had progressive dysphagia and dysarthria for the past few years. The patient is currently using a wheelchair for ambulation. She denied any sensory abnormalities, fluctuations in weakness, neck or back pain, shooting pain, bowel or bladder incontinence, muscle twitching, muscle pain, urine discoloration, double vision, or breathing difficulty.

**Figure 1:**
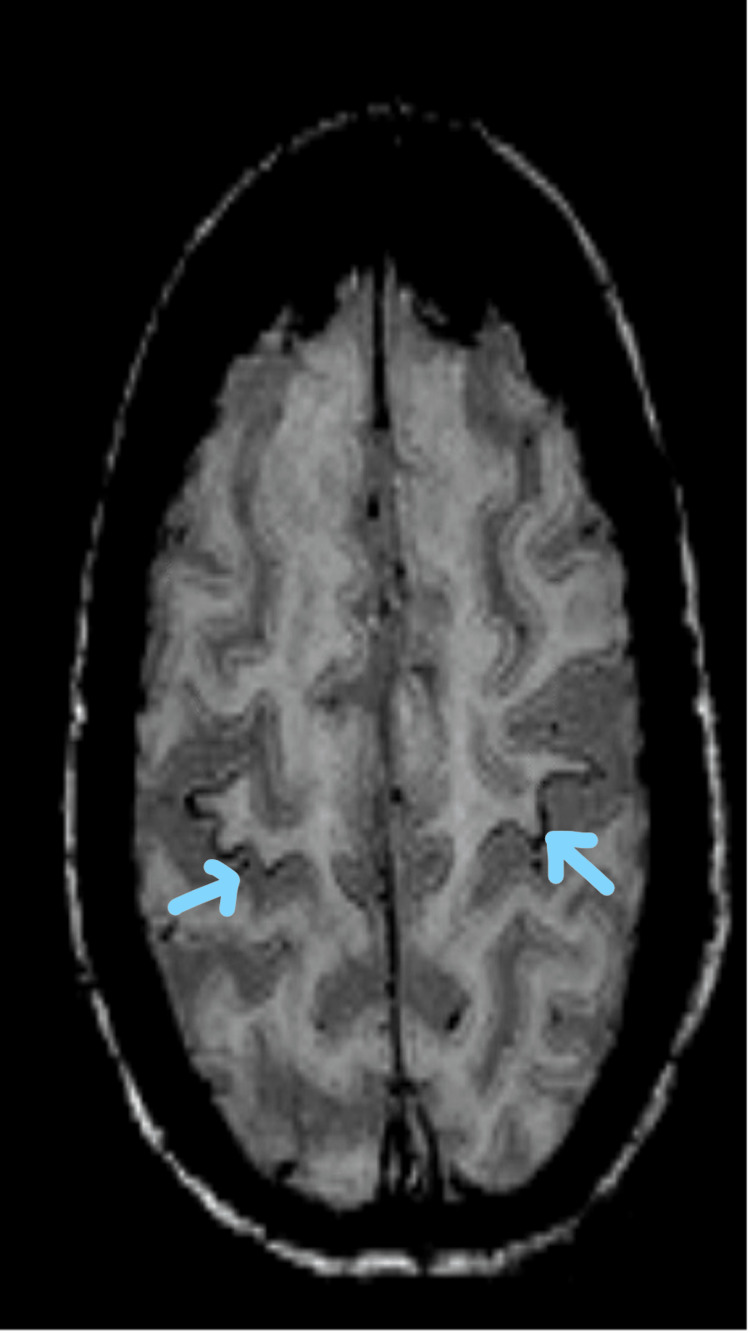
Motor band sign with bilateral hypointense signals in the pre-central region on SWI sequence in a patient with diagnosed PLS (blue arrows) SWI: susceptibility weighted imaging, PLS: primary lateral sclerosis

**Figure 2:**
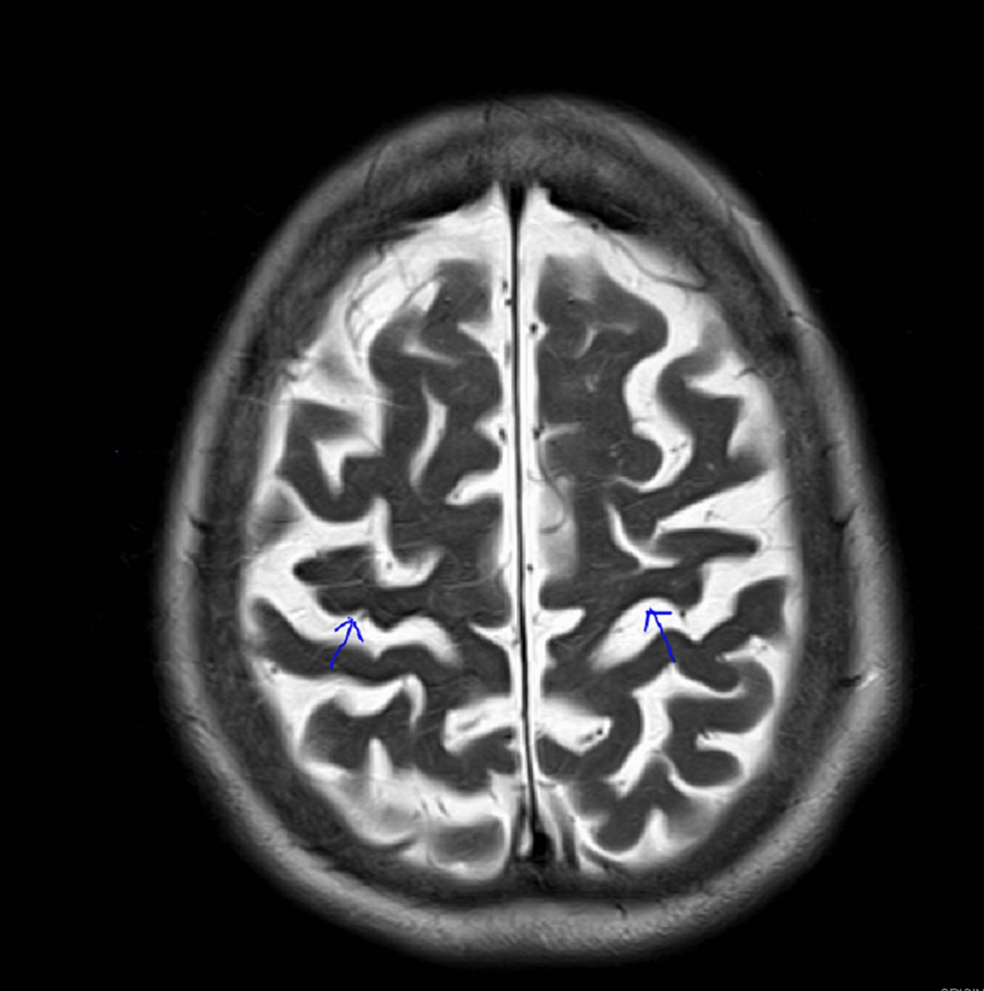
Bilateral hypointense signals on T2 sequence of brain MRI in the pre-central region (blue arrows) T2: transverse relaxation time, MRI: magnetic resonance imaging

Her examination was remarkable for impaired shrugging of shoulders bilaterally, suggesting spinal accessory nerve involvement. Based on the Muscle Research Grading System (MRGS) scale used to measure strength, she has a strength of 4 in bilateral upper extremities and lower extremities proximally, a strength of 4+ distally in bilateral upper extremities, and 4- in bilateral lower extremities. She has spasticity in her bilateral upper and lower extremities. Her deep tendon reflexes were exaggerated in the bilateral patellar, biceps, and triceps. She has a sustained ankle clonus bilaterally. Plantar reflex was mute, and Hoffman’s sign was positive bilaterally. Her sensory and cerebellar system examinations were completely unremarkable. EMG showed mild inactive chronic polyradiculopathy but was unremarkable for any lower motor neuron or sensory involvement in the upper and lower extremities. Based on consensus diagnostic criteria for PLS [1], the patient was diagnosed with primary lateral sclerosis. The patient’s MRI was significant for T2 and SWI sulcal hypointensities in the pre-central regions on the axial sequences bilaterally, suggestive of the “motor band sign” (Figure 1 and Figure 2). The patient was started on baclofen for spasticity. Her swallow evaluation showed aspiration potential for thin liquids, and her swallow function is being monitored periodically by the speech therapy team.

## Discussion

Primary lateral sclerosis was initially described by Jean-Martin Charcot in 1874 as “primary sclerosis of the lateral columns” [7]. Despite subsequent attempts to characterize the disease as a separate diagnostic entity, it was not until 1977 that this pathological entity attained renewed interest. Fisher [8] reported six cases of progressive weakness, with evidence of degeneration of the corticospinal tract without involving anterior horn cells. The diagnostic criteria for PLS have been evolving since the time they were initially proposed. The current diagnostic criteria have been based on clinical and laboratory features incorporated into the system with evolving experience in this area [1].

Laboratory features are majorly used to exclude the possibility of other diagnostic entities. There is currently no objective surrogate marker that is helpful in determining UMN involvement and aiding in the diagnosis of PLS.

The index patient had a progressive weakness with features of UMN involvement clinically. LMN involvement was excluded based on EMG. Given the clinical features of the upper motor neuron type of weakness in the index patient, the presence of the imaging feature of the motor band sign served as a surrogate marker for UMN involvement. Motor band sign, as previously described, is a hypointense signal in the motor cortex bilaterally on T2 and SWI sequences of brain MRI. This signal change is believed to be secondary to the degeneration of motor neurons in the primary motor cortex [4].

Motor band sign has been studied as a specific association with motor neuron disease such as PLS when there is UMN involvement. The presence of such findings in the index patient, who predominantly presented with UMN involvement, further supports the existing association. With the existing evidence, we propose motor band sign be considered as an imaging biomarker for upper motor neuron involvement in conditions such as PLS. However, further studies are needed to strongly establish this association.

## Conclusions

Despite being studied as a specific association, motor band sign has not been proposed as a surrogate marker for UMN involvement in ALS and PLS. Early recognition of this MRI pattern will allow the diagnosis of neurodegenerative diseases such as PLS to be made, especially when the clinical evaluation does not fulfill the existing diagnostic criteria. This in turn can improve the scope of early management strategies and prognostication, eventually translating into improving the quality of life.
